# Delayed pleuroperitoneal leak in an otherwise uncomplicated course of peritoneal dialysis

**DOI:** 10.1002/ccr3.7469

**Published:** 2023-06-13

**Authors:** Victor Shou Yee Kwan, Saskia Leibowitz

**Affiliations:** ^1^ Greater Brisbane Clinical School The University of Queensland Brisbane Queensland Australia; ^2^ Department of Nephrology Royal Brisbane and Women's Hospital Brisbane Queensland Australia

**Keywords:** peritoneal dialysis, peritoneal scintigraphy, pleural effusion, pleuroperitoneal leak

## Abstract

**Key Clinical Message:**

Pleuroperitoneal leaks are rare and normally arise as an early complication in peritoneal dialysis. This case illustrates the importance of considering pleuroperitoneal leaks as a cause for pleural effusions—even if peritoneal dialysis has been longstanding and uncomplicated.

**Abstract:**

A 66‐year‐old male on peritoneal dialysis for 15 months presented with dyspnoea and low ultrafiltration volumes. Chest radiography revealed a large right‐sided pleural effusion. Pleural fluid sampling and peritoneal scintigraphy confirmed a pleuroperitoneal leak.

Pleural effusions from pleuroperitoneal leaks are a rare but well‐recognized complication of peritoneal dialysis (PD). They normally arise early in the course of PD with 76% of reported cases occurring within 6 months and 89% within 12 months.^1^, ^2^, ^3^ Risk factors include congenital diaphragmatic defects, diaphragmatic muscular hypotonia, previous episodes of peritonitis, lymphatic drainage disorders, and polycystic kidney disease.^1^, ^2^, ^4^ Early identification of a pleuroperitoneal leak is essential as they may falsely mimic states of volume overload and lead to inappropriate treatment with higher osmolar dialysate fluids to increase ultrafiltration, potentially worsening the pleural effusion.^4^ We present a case of a late occurrence of a pleuroperitoneal leak.

A 66‐year‐old male on continuous ambulatory PD for 15 months in the context of interstitial nephritis presented with dyspnoea and low ultrafiltration volumes. Chest radiography revealed a large right‐sided pleural effusion (Figure 1A). Pleural fluid (PF) sampling via a chest drain confirmed a transudative effusion as per Light's criteria (PF protein/serum protein = 0.14, PF LDH/serum LDH = 0.23, PF LDH = 46 units/L). The PF white cell count was within normal limits at 85 × 10^6^/L, PF culture was negative, and no malignant cells were seen on cytology. The patient also had no systemic signs or symptomatology consistent with peritonitis or empyema. The PF glucose was 6.0 mmol/L and serum glucose was 5.7 mmol/L, yielding a PF to serum glucose gradient of 0.3 mmol/L and a PF to serum glucose ratio of 1.05. The patient's serum white cell count, serum albumin, and cardiac status were within normal limits.

**FIGURE 1:**
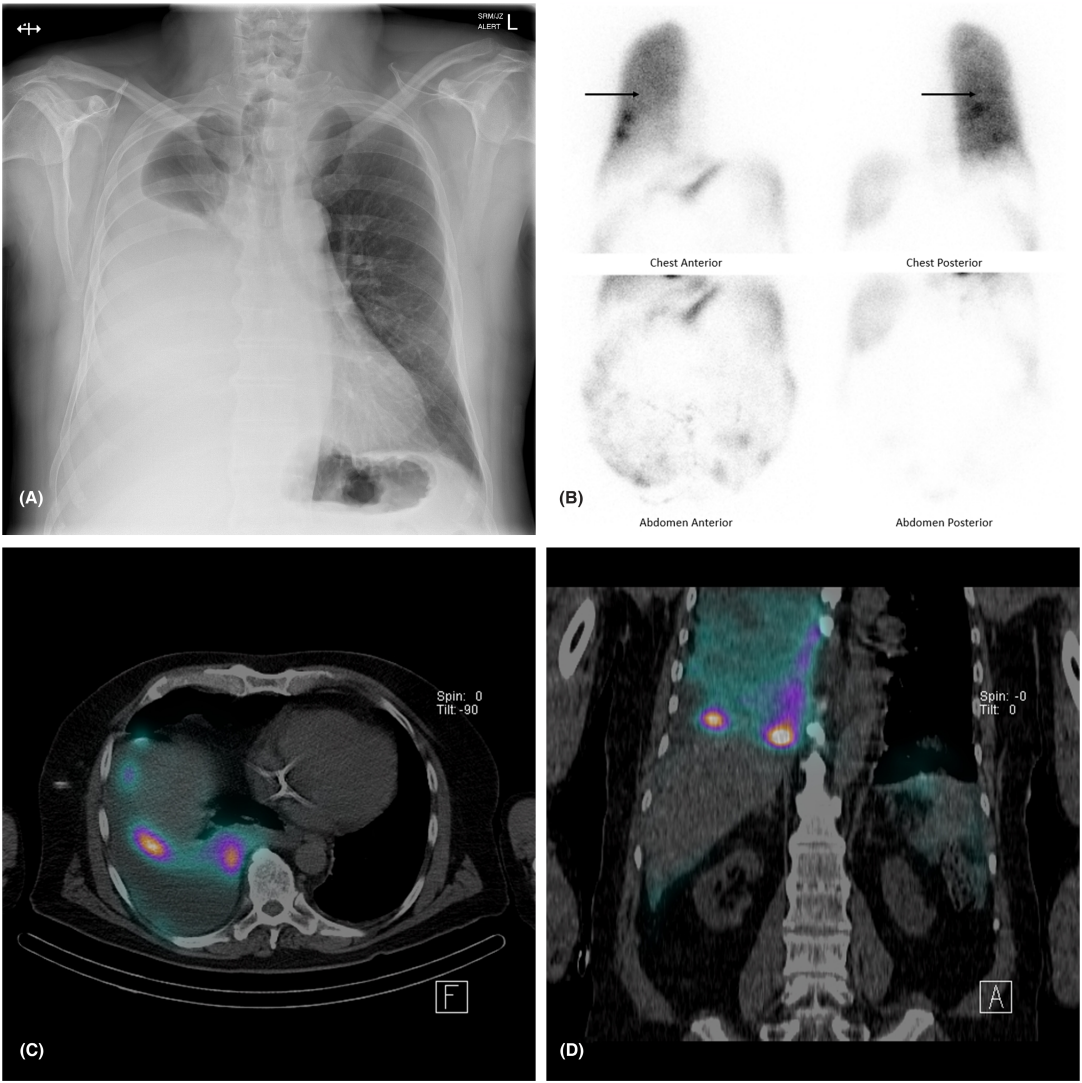
(A) Posterior–anterior chest radiograph demonstrating a large right‐sided pleural effusion. (B) Peritoneal scintigraphy planar images at 1 h following administration of dialysate fluid containing 111 MBq Tc‐99m macroaggregated albumin. Pleuroperitoneal communication is observed with radiotracer activity diffusely within the peritoneal cavity and right hemithorax (arrows). (C, D) Axial and coronal sections of SPECT–CT display right‐sided pleural effusion accumulated with radiotracer and multiple foci along the right hemidiaphragm. A clear fistula was not identifiable. Tunneled hemodialysis catheter in situ.

With an unclear etiology, the working diagnosis was a pleuroperitoneal leak secondary to an acquired diaphragmatic defect despite no known risk factors and an uncomplicated course of PD. A peritoneal scintigraphy was performed by administering dialysate fluid containing 111 MBq technetium‐99m macroaggregated albumin via the Tenckhoff catheter. The chest drain was clamped prior to administration. Planar images and SPECT–CT at 1 h were consistent with a pleuroperitoneal leak but with no macroscopic defect identifiable (Figure 1B–D). As a result, the surgical team concluded the patient would not be suitable for ongoing PD. The pleural effusion was drained, PD was withdrawn, and the patient was converted to hemodialysis.

Pleural effusions are common in PD patients and can be life‐threatening—thus, the etiology should be promptly determined. Definitive diagnosis of pleuroperitoneal leaks often relies on both biochemical and radiological assessment.^4^ While some propose a PF to serum glucose gradient of at least 2.8 mmol/L is highly suggestive (not satisfied in this case), others postulate any PF to serum glucose ratio >1.0 is suggestive.^1^, ^2^, ^3^ Analysis with peritoneal scintigraphy carries a sensitivity of up to 50%.^1^ This case of a pleuroperitoneal leak 15 months after commencing dialysis highlights that clinicians should maintain a high index of suspicion for a pleuroperitoneal leak in the face of a unilateral pleural effusion even late in the course of uncomplicated PD.

## AUTHOR CONTRIBUTIONS

**Victor Shou Yee Kwan:** Resources; writing – original draft; writing – review and editing. **Saskia Leibowitz:** Conceptualization; supervision; writing – original draft; writing – review and editing.

## FUNDING INFORMATION

No financial support was sought out for this case image report.

## CONFLICT OF INTEREST STATEMENT

There are no potential conflicts of interest to be declared by the authors.

## CONSENT

Written informed consent was obtained from the patient to publish this report in accordance with the journal's patient consent policy.

## Data Availability

No data sets were generated or analyzed for this case report.
